# Encapsulation of Microalgae *Tisochrysis lutea* Extract in Nanostructured Lipid Carriers (NLCs) and Evaluation of Their Sunscreen, Wound Healing, and Skin Hydration Properties

**DOI:** 10.3390/md22110487

**Published:** 2024-10-30

**Authors:** Natalia Chatzopoulou, Chrysi Chaikali, Eleni Mourkogianni, Constantinos M. Mikelis, Vasilis Andriopoulos, Michael Kornaros, Konstantinos Avgoustakis, Fotini N. Lamari, Sophia Hatziantoniou

**Affiliations:** 1 Laboratory of Pharmaceutical Technology, Department of Pharmacy, School of Life Sciences, University of Patras, 26 504 Rion, Greecechem3813@ac.upatras.gr (C.C.); avgoust@upatras.gr (K.A.); 2 Laboratory of Molecular Pharmacology, Department of Pharmacy, School of Life Sciences, University of Patras, 26 504 Rion, Greece; eleni9119@yahoo.gr (E.M.); kmikelis@upatras.gr (C.M.M.); 3 Laboratory of Biochemical Engineering & Environmental Technology (LBEET), Department of Chemical Engineering, University of Patras, 26 504 Patras, Greece; billandri@upatras.gr (V.A.); kornaros@chemeng.upatras.gr (M.K.); 4 Laboratory of Pharmacognosy and Chemistry of Natural Products, Department of Pharmacy, School of Life Sciences, University of Patras, 26 504 Rion, Greece; flam@upatras.gr

**Keywords:** *Tisochrysis lutea* extract, nanostructured lipid carriers, SPF, antioxidant, skin hydration, wound healing

## Abstract

Traditional sunscreens have relied on synthetic compounds to protect against harmful ultraviolet (UV) radiation. However, there is increasing interest in utilizing the natural photoprotective properties of microalgae extracts. This approach does not only aim to enhance the stability and efficacy of sun protection formulae but also seeks to reduce the reliance on synthetic sunscreens. This study investigates the encapsulation of *Tisochrysis lutea* extract (TL) in nanostructured lipid carriers (NLCs) to create a combination (NLC-TL) with enhanced physicochemical stability, antioxidant activity, SPF efficacy, wound healing capacity, and skin hydration. The particle size and ζ-potential were approximately 100 nm and −50 mV, respectively, and both formulations successfully passed the stability tests. The antioxidant activity, measured via DPPH assay, revealed that NLC-TL achieved the highest free radical scavenging activity across all tested concentrations, indicating a synergistic effect. The incorporation of TL in NLCs maintained the sun protection factor (SPF) of a 2% extract solution (1.53 ± 0.13). The wound healing assay indicated that NLC-TLs significantly enhanced wound closure compared to controls and TL alone. Additionally, skin hydration tests on healthy volunteers revealed that NLC-TLs provided superior and sustained hydration effects. These results highlight NLC-TLs’ potential as a multifunctional topical agent for cosmetic and therapeutic applications.

## 1. Introduction

Microalgae have gained increasing attention in recent years due to their remarkable versatility and potential applications across various industries, particularly in cosmetics and pharmaceuticals [1,2,3,4,5]. Among these microalgae, *Tisochrysis lutea* stands out as a promising candidate, having a rich reservoir of bioactive compounds, both hydrosoluble and liposoluble, such as fucoxanthin, phenolic substances, and lipids, which hold promise for a variety of topical applications [6,7,8,9,10]. The distinctive biochemical composition of *T. lutea* not only offers cosmetic benefits but also holds therapeutic potential, rendering it an attractive resource for the development of novel skincare and pharmaceutical products [5,11].

*Tisochrysis lutea*, formerly known as *Isochrysis galbana*, is a marine microalga that possesses distinctive features that contribute to its industrial significance [12]. Rich in essential fatty acids, vitamins, antioxidants, and other bioactive molecules, *T. lutea* exhibits properties highly coveted for promoting skin health and overall well-being [13,14,15].

The bioactive compounds present in *T. lutea* have demonstrated antioxidant and anti-inflammatory properties, essential for maintaining skin health and combating aging-related skin issues. Moreover, the unique lipid profile of *T. lutea*, including omega-3 fatty acids, such as docosahexaenoic acid (DHA) and eicosapentaenoic acid (EPA), makes it an ideal candidate for formulations aimed at enhancing skin barrier function and promoting tissue regeneration [5,16,17]. Furthermore, previous studies have highlighted *T. lutea* extract’s enhanced ability to absorb UV radiation [9].

In addition to its cosmetic applications, *T. lutea* holds promise in pharmaceutical formulations for the topical treatment of various skin conditions, including eczema, psoriasis, and acne. Investigations into the anti-inflammatory and antimicrobial activities of *T. lutea* extracts have underscored their potential efficacy in alleviating symptoms associated with these dermatological disorders [5,18]. Moreover, the capacity of *T. lutea* to modulate immune responses and protect from UV-induced damages further underscores its therapeutic relevance in pharmaceutical formulations that promote wound healing [5,19].

Despite the growing interest in *T. lutea* for cosmetic and pharmaceutical applications, there remains a need for comprehensive studies elucidating its mechanisms of action and efficacy in different formulations.

Lipid nanoparticles have emerged as versatile carriers for the encapsulation and delivery of bioactive compounds, owing to their biocompatibility, stability, and ability to enhance the solubility and bioavailability of hydrophilic and hydrophobic substances [20,21,22,23,24]. Nanostructured lipid carriers (NLCs), a subclass of lipid nanoparticles, represent a sophisticated approach in drug delivery systems, characterized by their unique structure comprising a blend of solid and liquid lipids. This structural arrangement offers a superior drug loading capacity, controlled release kinetics, and enhanced stability compared to conventional lipid nanoparticles [25,26,27].

The incorporation of *T. lutea* extract into lipid nanoparticles, particularly NLCs, holds immense potential for harnessing the therapeutic and functional benefits of this microalga in various applications. By incorporating *T. lutea* extracts within lipid nanoparticles, researchers could overcome challenges associated with the delivery of microalgae-based products, including stability, bioavailability, and controlled release.

Utilizing NLCs as carriers for *T. lutea* extracts offers several advantages. The nanostructured lipid matrix provides a protective environment, shielding the bioactive compounds from degradation and oxidative stress, thereby preserving their integrity and potency. Additionally, the tunable properties of NLCs enable precise control over the release kinetics of *T. lutea* components, facilitating sustained and targeted delivery. Moreover, the biocompatibility of lipid nanoparticles ensures the compatibility with biological systems, minimizing potential adverse effects and enhancing the safety profile of *T. lutea*-based formulations. Despite the advantages that formulating in nanocarriers may provide, the incorporation of *T. lutea* extracts has not been studied so far.

This work aimed to achieve the encapsulation of *T. lutea* extract (TL) into NLCs and to explore its impact on the sun protection factor (SPF). This study encompasses the preparation of nanostructured lipid carriers for encapsulating *T. lutea* extract, alongside empty nanocarriers for comparison. The subsequent physicochemical characterization of the nanocarriers, stability assessments, and evaluation of radical scavenging capacity were conducted. Additionally, the in vitro SPF determination using Mansur’s equation and in vivo assessment of skin hydration in healthy volunteers were performed to ascertain the efficacy and safety of the formulated NLCs.

Based on the findings of this study, the applicability of the strategy involving *T. lutea* extract encapsulated in NLCs for developing sunscreen and skin care products enriched with natural ingredients was verified, thereby paving the way for innovative approaches in skincare and pharmaceutical formulations.

## 2. Results and Discussion

### 2.1. T. lutea Extract Characterization

Immediately after the preparation of the extract, the chlorophyll a and total carotenoid and xanthophyll contents were 194.35 ± 4.39 mg/L extract and 93.53 ± 2.27 mg/L extract, respectively, while the reactivity towards the FC assay was 71.95 ± 1.29 mg GAE/L extract.

An HPLC-DAD analysis of the extract confirmed the abundance of xanthophylls and chlorophyll a (Figure S1). Taking into account the peak area percentage at 430 nm, the major compound (40%) was the allenic monoepoxide xanthophyll, fucoxanthin, followed by chlorophyll a (17%) and phaeophytin a (11.7%), in accordance with previous studies [28]. Other three allenic trans-xanthophylls, one allenic cis-xanthophyll, two all-trans xanthophylls, and one cis-xanthophyll were also present in significant amounts. The only representative of carotenes was carotene-b. Additionally, two phenolic acid derivatives eluted early at 1.82 and 3.43 min with a maximum absorbance at 275 and 265 nm, respectively.

### 2.2. Physicochemical Characteristics of NLCs and NLC-TLs

**Figure 1:**
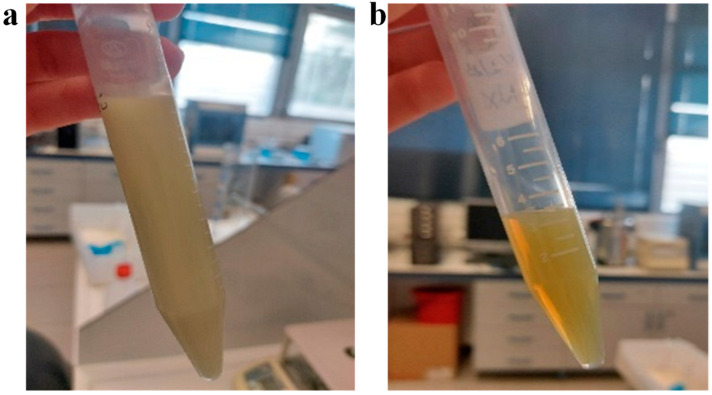
Photographs of NLC-TLs (**a**) before and (**b**) after probe sonication.

After their preparation, the samples were visually inspected. The NLCs and NLC-TLs were translucent and homogeneous. NLCs were off-white while NLC-TLs were greenish due to the presence of TL. Both NLCs and NLC-TLs exhibit the characteristic iridescence and optical clarity due to the size of the particles in the nanoscale (Figure 1). This phenomenon is called the “Tyndall effect” and confirms the existence of nanoparticles in the sample. It is one of the key optical features of nanocolloids and was named after the 19th-century British physicist John Tyndall and resulting from the scattering of light by the colloidal nanoparticles [29].

One day after preparation, the physicochemical characteristics of both carriers were assessed. The mean size of the NLCs was 99.83 nm ± 4.01 nm, with the PDI 0.264 ± 0.004 and the ζ-potential −46.9 mV ± 1.1 mV. The values of NLC-TL particles were 103.88 nm ± 7.14 nm, 0.255 ± 0.003, and −46.7 mV ± 7.4 mV, respectively. The content of *T. lutea* extract in NLC-TLs was assessed using spectrophotometry based on its chlorophyl content (655 nm) [30]. The TL content of NLC-TLs was found to be 9.7 mg/ mL ± 3.1 mg/ mL with the LC% (loading) 0.64 ± 0.22 and the EE% 66.0 ± 30.3.

### 2.3. Stability of NLCs and NLC-TLs

**Figure 2:**
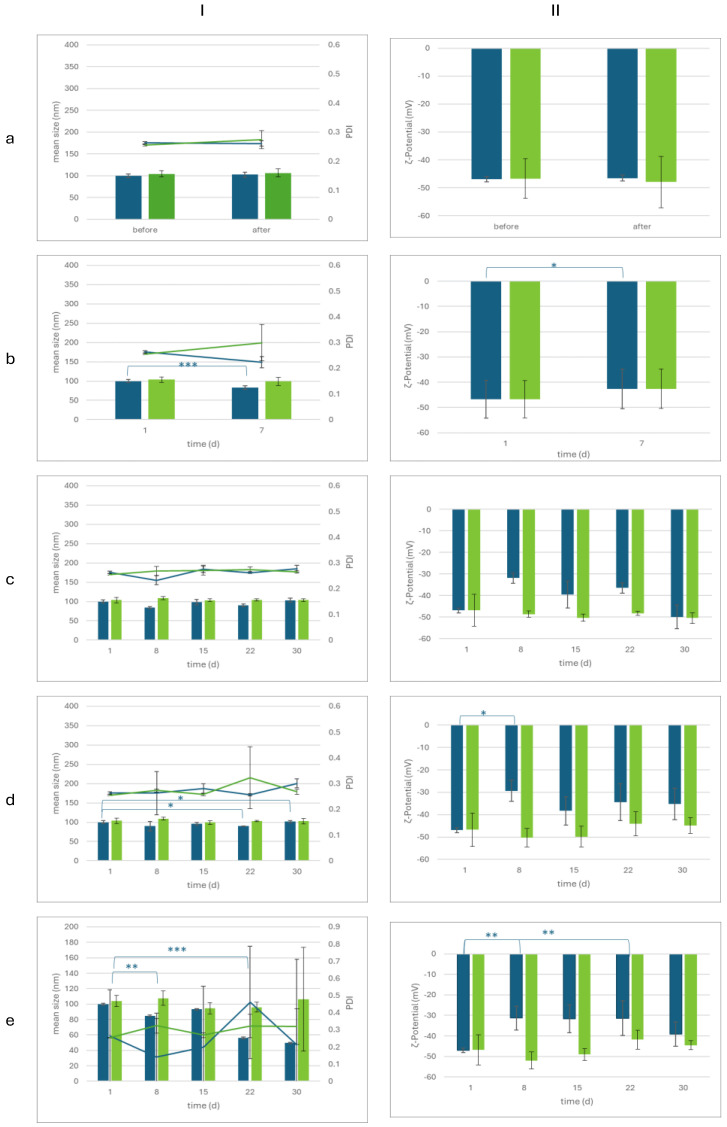
Monitoring of (**I**) size distribution (size: bar, PDI: line) and (**II**) ζ-potential of NLC (blue) and NLC-TL (green), after (**a**) centrifugation, and (**b**) accelerated aging and storage for 90 days at (**c**) 4 °C, (**d**) 25 °C, and (**e**) 40 °C, 75% RH. * *p* < 0.05, ** *p* < 0.01, *** *p* < 0.005.

Following sample preparation, the formulations were subjected to centrifugation to determine their stability under mechanical stress and an accelerated aging test to assess their long-term stability (Table S1). Post-centrifugation, both the size, polydispersity index (PdI), and ζ-potential of NLCs and NLC-TLs remained stable, as the differences in values before and after centrifugation were not statistically significant (*p* > 0.05) (Figure 2a).

At the end of the accelerated aging test, as depicted in Figure 2b, a statistically significant reduction in the size and ζ-potential of NLCs was observed (*p* < 0.05). This change in both size and ζ-potential after the accelerated aging test may be indicative of the poor long-term stability of the lipid nanocarriers (NLCs). The storage at 4 °C of both NLCs and NLC-TLs for 30 days kept stable all their physicochemical characteristics (size, PDI, and ζ-potential) (*p* > 0.05, Figure 2c). During storage at 25 °C, the mean size of NLCs presented fluctuations from the 22nd day (*p* < 0.05) and the ζ-potential was significantly reduced at the 8th day (*p* < 0.05). The physicochemical characteristics of NLC-TLs, on the contrary, remained stable (*p* > 0.05) for at least 30 days of storage at 25 °C (Figure 2d). The average size and ζ-potential values of the NLC samples significantly decreased after the 8th day of storage at 40 °C, 75% RH. PDI remained stable despite some fluctuations over the 30 days (*p* > 0.05). The NLC-TL samples did not exhibit significant differences in mean droplet size, PDI, and ζ-potential values at least for the 30-day storage (*p* > 0.05) (Figure 2e).

### 2.4. Antioxidant Activity of NLC-TLs

**Figure 3:**
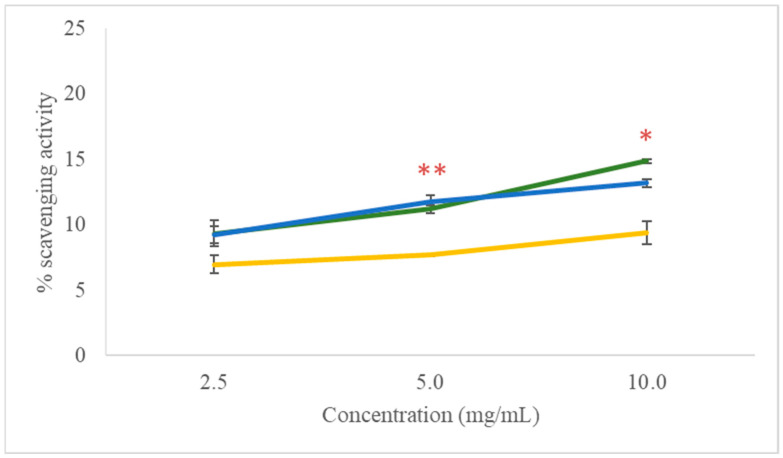
Free radical scavenging activity of NLC-TL (green), NLC (blue), and TL (yellow) as a percentage of scavenging activity. * *p* < 0.05, ** *p* < 0.01.

The antioxidant activity, specifically the free radical scavenging activity, of the NLC-TL, NLC, and TL is illustrated in Figure 3. This activity was quantified using the DPPH assay and is expressed as a percentage of scavenging activity at three different concentrations (10 mg/mL, 5 mg/mL, and 2.5 mg/mL).

The results indicate that the NLC-TL consistently exhibited the highest antioxidant activity across all concentrations, with free radical scavenging activity values of 14.857 ± 0.156% at 10 mg/mL, 11.189 ± 0.330% at 5 mg/mL, and 9.318 ± 0.990% at 2.5 mg/mL. The NLC alone also exhibited antioxidant activity, although slightly lower than the NLC-TL, with corresponding values of 13.177 ± 0.330%, 11.739 ± 0.508%, and 9.208 ± 0.660% at the respective concentrations. In contrast, TL displayed the lowest activity, with 9.391 ± 0.890% at 10 mg/mL, 7.667 ± 0.110% at 5 mg/mL, and 6.970 ± 0.660% at 2.5 mg/mL. The scavenging activity of ascorbic acid, used as a positive control, reached 97.818 ± 0.822% at the highest concentration (2 mg/mL) and 8.483 ± 0.435% at lower concentrations (0.005 mg/mL). The differences observed were statistically significant (*p* < 0.05) when compared to 10 mg/mL and 5 mg/mL of the NLC-TL and NLC. Overall, the combination of the NLC with TL (NLC-TL) appears to enhance the antioxidant activity compared to the NLC or TL alone, particularly at the higher concentrations tested, although the overall antioxidant activity remained relatively low.

### 2.5. Evaluation of SPF In Vitro

**Figure 4:**
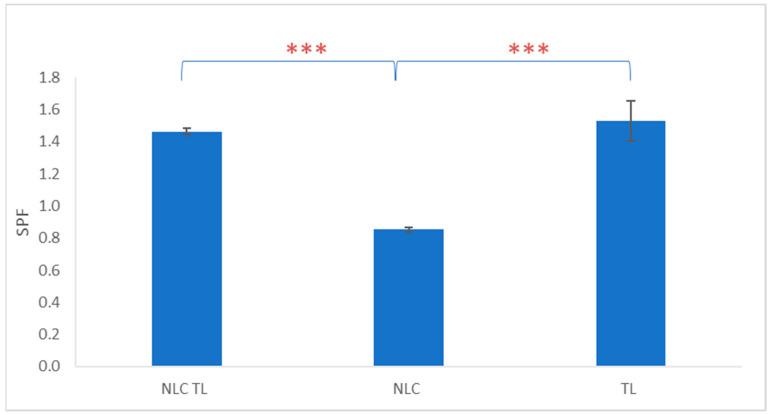
SPF calculated in vitro for NLC-TL, NLC, and *T. lutea* extract (TL). *** *p* < 0.001.

The sun protection factor (SPF) of NLC-TLs, as assessed by UV–Vis spectroscopy (Figure S2), was 1.46 ± 0.02 and it was similar (*p* > 0.05) to the 1.53 ± 0.13 SPF value for TL at a TL concentration corresponding to the NLC-TL content. This suggests that the encapsulation of *Tisochrysis lutea* (TL) into the NLC did not significantly alter its SPF potential, highlighting the efficacy of the encapsulation process in maintaining the active properties of TL (Figure 4, Table S2). In contrast, the SPF of the NLC at the same concentration as NLC-TL was found to be 0.853 ± 0.014, which is significantly lower (*p* < 0.050) compared to the SPFs of both the TL and NLC-TL.

### 2.6. Cell Viability

**Figure 5:**
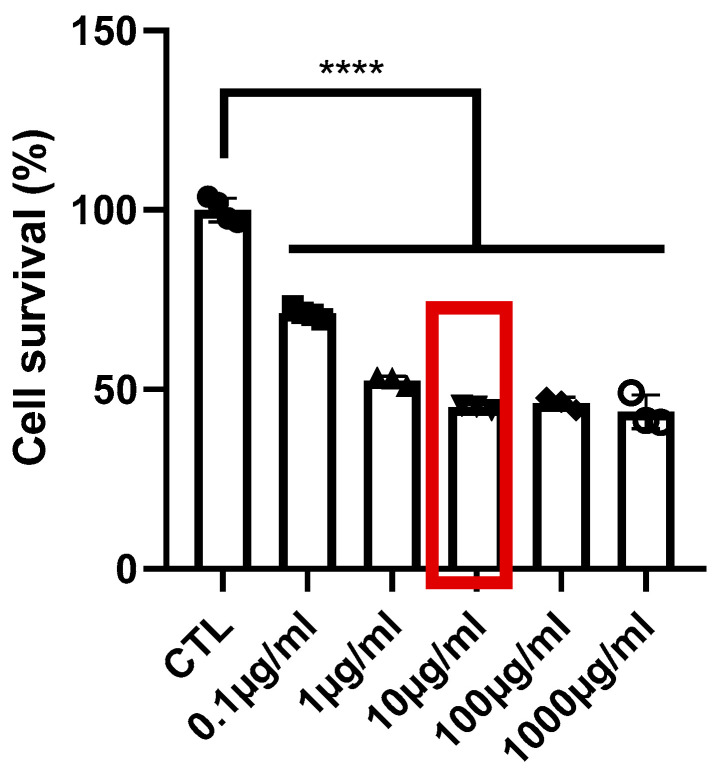
Evaluation of different doses of the TL compound on NIH3T3 cell survival. The red box indicates the concentration of TL used for wound healing assay. **** *p* < 0.0001.

We first performed a dose–response experiment to identify the effect of increasing TL doses on NIH3T3 fibroblast survival (Figure 5). Proliferation was significantly decreased from the lowest dose tested (0.1 μg/mL); however, the cells looked healthy with no signs of stress up to the dose of 10 μg/mL. Signs of morphological alterations of NIH 3T3 fibroblasts (early cell death) were observed at doses 100 μg/mL and 1000 μg/mL; thus, we worked with the highest tested concentration at which the cells looked healthy (10 μg/mL).

### 2.7. Wound Healing Assay

**Figure 6:**
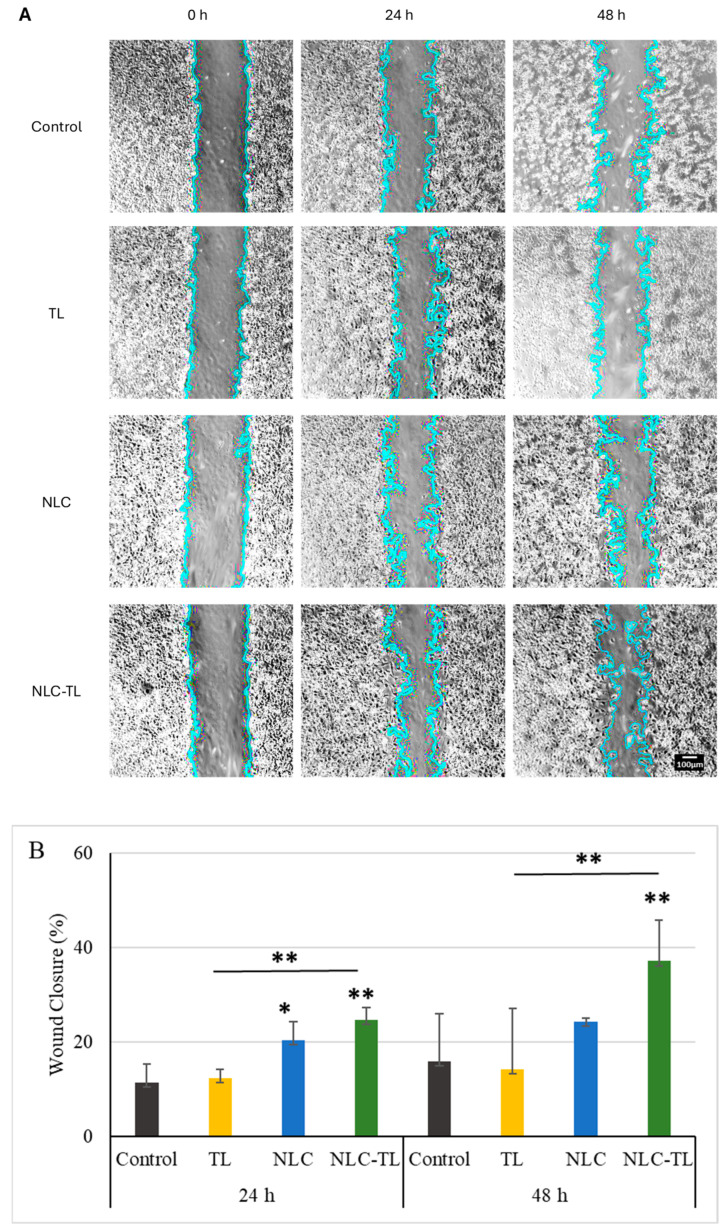
Wound healing assay in the presence or absence (control) of TL, NLC, and NLC-TL. Evaluation of the effect of the 10 μg/ mL of the TL compound, NLC, and NLC-TL on wound healing on NIH3T3 cells: (**A**) representative images at predefined time points (0 h, 24 h, and 48 h) and (**B**) quantifications of the wound closure. Results are presented as mean ± SD. * *p* < 0.05; ** *p* < 0.01.

To define the effect of the TL, NLC, or NLC-TL treatment on wound closure, we conducted an in vitro wound healing assay on NIH 3T3 fibroblasts and assessed the percentage of the scratch area reduction at 24 h and 48 h (Figure 6). The treatment with 10 μg/ mL of TL did not affect the wound closure neither at 24 h (12.30% ± 6.35%) nor at 48 h (14.22% ± 10.87%), compared to the corresponding controls (11.43% ± 4.638% at 24 h and 15.94% ± 8.296% at 48 h) (Figure 6B). Incubation with the empty NLC significantly reduced the scratch area at 24 h (20.39% ± 3.485%, *p* < 0.05), a phenomenon not further observed at 48 h (24.28% ± 4.302%) (Figure 6B). However, the same concentration of the TL-loaded NLC resulted in a statistically significant induced wound closure at both 24 h (24.70% ± 6.925%, *p* < 0.01) and 48 h (37.12 ± 11,48%, *p* < 0.01) compared to the control and TL treatment groups (Figure 6B), demonstrating that NLC encapsulation induced TL efficacy on the fibroblast wound closure in vitro.

### 2.8. Skin Hydration Effect of NLC-TLs

The impact of NLCs and NLC-TLs on skin hydration was evaluated in a cohort of healthy volunteers. Following application, significant enhancements in skin hydration were observed at 30 min post-application for both the NLC and NLC-TL formulations.

Specifically, the skin hydration level 30 min post-application of the NLC was 37.0 ± 8.8 units, representing an increase of 9.4 ± 6.1 units compared to the baseline (27.6 ± 7.9 units). This enhancement in skin hydration remained consistent up to 120 min post-application, indicating the sustained efficacy of NLCs in maintaining skin hydration.

Similarly, the application of the NLC-TL resulted in a comparable enhancement in skin hydration at 30 min, with skin hydration levels reaching 37.3 ± 9.3 units and an increase of 8.8 ± 6.2 units compared to baseline.

Throughout the experiment, untreated skin demonstrated stable hydration levels, with no statistically significant alterations observed (*p* > 0.05). The hydration levels of untreated skin averaged 28.4 ± 9.3 units.

**Figure 7:**
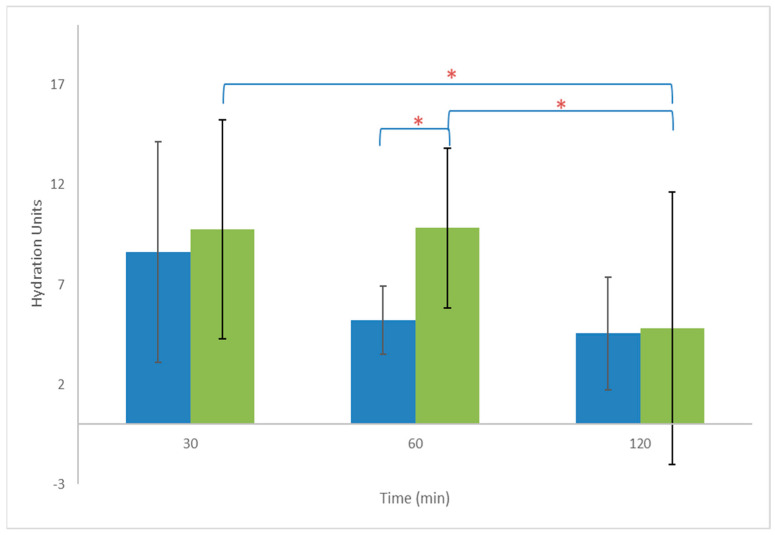
Monitoring of skin hydration change (normalized values) up to 120 min post treatment with NLC (■) and NLC-TL (■). * *p* < 0.05.

Normalized hydration values, obtained by subtracting the hydration change of untreated skin from the corresponding values of treated skin, are depicted in Figure 7.

At one hour post-application, the skin hydration change induced by the NLC-TL was significantly higher (*p* < 0.05) compared to the NLC (9.0 ± 4.9 units and 5.2 ± 1.7 units, respectively). However, by the 120 min mark, the skin hydration changes became similar for both formulations (NLC-TL: 5.9 ± 2.8 units and NLC: 4.5 ± 2.8 units, *p* > 0.05), suggesting convergence in their hydration effects over time.

## 3. Discussion

The present study successfully encapsulated *Tisochrysis lutea* extract (TL) within nanostructured lipid carriers (NLCs), resulting in a formulation (NLC-TL) that demonstrates significant potential for skin protective applications. The findings suggest that the incorporation of TL into NLCs not only provides distinct physical and chemical properties but also enhances the functional performance of the TL.

The physicochemical characterization of the NLC and NLC-TL formulations revealed that both systems exhibit desirable nanoparticle characteristics, including particle sizes within the optimal range for topical delivery (around 100 nm), low polydispersity indices (PDI < 0.3), and high absolute values of ζ-potential, indicative of good colloidal stability due to electrostatic repulsion, a key factor in preventing aggregation [31,32]. These results are consistent with the findings of Ashfaq et al. (2023) [33], who reported that lipid-based nanoparticles within comparable size ranges exhibit enhanced stability and the bioavailability of encapsulated bioactive compounds. The slight differences in particle size and the polydispersity index (PDI) between NLCs and NLC-TLs can be attributed to the incorporation of TL, which slightly increased the particle size but did not significantly alter the PDI or ζ-potential, maintaining the overall stability of the nanocarriers. The ζ-potential of NLCs and NLC-TLs was measured at −46.9 mV and −46.7 mV, respectively, indicating no significant change in the surface charge after the encapsulation of TL. This can be explained by the dominant negative charge imparted by the lipid matrix in the NLC formulation, which contains lecithin [27,31]. Since TL is encapsulated within the NLC core, rather than being adsorbed on the surface, its addition did not significantly affect the surface charge of the NLC particles. Both formulations maintain excellent colloidal stability, as evidenced by ζ-potential values well below −30 mV.

Furthermore, the NLC-TL formulation’s greenish hue, attributed to the presence of the TL, did not adversely affect the optical clarity of the formulation, which is essential for consumer acceptance in cosmetic applications. The observation of the Tyndall effect further confirms the nanoscale nature of the carriers, which is in agreement with previous studies utilizing similar methodologies for nanoparticle characterization [34].

The stability assessments, including centrifugation, accelerated aging, and storage at various temperatures, indicate that the NLC-TL formulation possesses superior long-term stability compared to NLC. Notably, the NLC-TL maintained its physicochemical properties without significant degradation even at 40 °C and 75% relative humidity, a condition under which the plain NLC exhibited marked instability. This stability can be attributed to the interaction between the TL and the lipid matrix, which likely provides additional structural integrity to the nanocarriers, as suggested by similar studies [31,35].

The enhanced free radical scavenging activity observed in the NLC-TL formulation compared to free TL is a significant finding, although the increase in antioxidant activity was modest. This enhancement can be attributed to the improved dispersion and protection of the bioactive compounds within the NLC matrix, which helps prevent premature degradation and enhances their interaction with free radicals. These findings are corroborated by other researchers, who demonstrated that lipid nanoparticles could significantly enhance the antioxidant efficacy of encapsulated natural compounds by protecting them from oxidative degradation and improving their bioavailability [33,36].

While the DPPH assay indicates that the NLC-TL exhibits slightly higher antioxidant activity than the NLC alone, the incorporation of TL in NLCs serves broader purposes beyond the direct enhancement of the antioxidant potential. TL contains bioactive compounds, particularly carotenoids like fucoxanthin, which are known to provide multiple benefits in skincare, including photoprotection, the enhancement of skin moisture, elasticity, and overall skin structure [37]. In our study, we demonstrate significant improvements in UV protection, fibroblast wound healing, and the in vivo enhancement of skin hydration. Antioxidant activity is only one aspect of their beneficial properties, further demonstrating the versatility of TL beyond its antioxidant properties.

The encapsulation of extracts within nanostructured lipidic carriers offers additional advantages, such as the enhanced stability of bioactive compounds, improved bioavailability, and sustained release over time, which may lead to long-term antioxidant effects not fully captured by short-term assays like DPPH [38]. Furthermore, although the free radical scavenging activity of the NLC alone was notable, the combination with TL resulted in a more balanced and sustained response across multiple concentrations. These findings suggest that the NLC system not only serves as a protective carrier but also potentially enhances the biological activity of encapsulated compounds, which has been corroborated by other studies on microalgae-based bioactive compounds [33,39].

While the official method for estimating the SPF for sun protection products is performed in healthy volunteers [40], a preliminary SPF assessment can also be conducted in vitro using spectrophotometry [41]. The SPF evaluation revealed that, while the TL and NLC-TL exhibited some degree of UV protection, the values were relatively low, suggesting that the primary benefit of the TL in this formulation may lie elsewhere, such as in antioxidant or skin hydration effects rather than direct UV blocking. Although microalgae extracts are known to provide photoprotection mainly because of the presence of UV-absorbing compounds [42], the strain and the extraction method should be taken into consideration in order to possess the full range of multifunctional skin benefits.

The biocompatibility and low cytotoxicity of the formulations were confirmed by cell viability assays. In terms of cell viability, the results indicated that the TL in higher concentrations could induce cytotoxicity, highlighting the importance of optimizing the dosage for therapeutic applications. The use of NLCs mitigated this effect, suggesting that encapsulation could provide a controlled release mechanism, reducing the risk of cytotoxicity, while maintaining efficacy. This observation aligns with Patel et al. (2024), who emphasized the role of lipid nanocarriers in modulating the release profiles of potent bioactive compounds, thereby enhancing their safety and therapeutic index [43].

The wound healing assay demonstrated that NLC-TL significantly improved wound closure rates compared to both the free TL and plain NLC, particularly at 48 h post-treatment. This enhanced efficacy could be attributed to the improved delivery and sustained release of the TL from the NLC matrix, allowing for prolonged biological activity at the site of application. These findings are consistent with the previously reported results stating that encapsulation within lipid nanoparticles could enhance the wound healing properties of bioactive compounds by facilitating their sustained release and deeper penetration into the skin [27,33].

Additionally, the skin hydration studies indicated that both the NLC and NLC-TL formulations effectively increased skin hydration, with the NLC-TL providing a more prolonged effect. The significant enhancement in skin hydration observed after the application of the NLC-TL compared to NLC alone suggests that the TL plays a critical role in maintaining skin moisture, potentially due to its bioactive compounds that interact favorably with the skin barrier, findings that are consistent with previous studies by our group [9] and other researchers, such as Ashfaq et al. (2023) [33], who reported improved hydration effects when using lipid-based carriers for the delivery of natural extracts. This is particularly relevant for dermatological applications where sustained hydration is crucial for maintaining skin health.

## 4. Materials and Methods

### 4.1. Materials

The following ingredients used for the preparation of NLC were of cosmetic grade: saboderm TCC (SABO S.p. A., Bergamo, Italy), softisan 100 (Sasol GmbH, Hamburg, Germany), solutol HS 15 (BASF, Ludwigshafen, Germany), and emulmetik 900 (Lucas Meyer Cosmetics, Champlan, France). All other materials were of analytical grade: water for injection (WFI) (Demo S.A., Pharmaceutical Industry, Kryoneri, Attica, Greece), and ethanol (absolute, purity ≥ 99.8%) (Acros Organics, Geel, Belgium).

#### *T. lutea* Extract (TL) Preparation and Characterization

The *T. lutea* extract was prepared at the Chemical Engineering Department of the University of Patras as previously described [15,44]. Briefly, *T. lutea* was cultivated for 24 days in Erlenmeyer flasks of 500 mL capacity and with operational volume of 400 mL using four times concentrated f/2 medium without silicate at 25 °C and illumination (6000 K) provided from below at ~350 μmol m^−2^ s^−1^. Mixing and CO_2_ were provided by aeration with ambient air at ~2.8 vvm. Initial biomass concentration was 0.1–0.16 g L^−1^ DW. Microalgal biomass was collected with centrifugation at 3780× *g* for 7 min, washed with isotonic ammonium bicarbonate solution, and freeze-dried. Approximately 20 mg of freeze-dried biomass (moisture content ~4.7%, and ash content ~2% DW) was transferred to an Eppendorf tube with 2 mL of pure ethanol. The tube was placed in an ultrasonic bath (BioLine Scientific, Athens, Greece) where extraction took place for 15 min and temperature maintained below 40 °C [44]. The extract was stored at 4 °C sealed in a glass container.

The extract was characterized immediately after its preparation. Chlorophyll a, and total carotenoids and xanthophylls were measured spectrophotometrically (UV-1800 UV–Vis Spectrophotometer, SHIMADZU, Kyoto, Japan) [45], while the reactivity towards the Folin–Ciocalteau (FC) assay was measured as previously described [15,44].

Fingerprinting analysis of *T. lutea* extract was performed by HPLC-DAD using a Poroshell C18 column (250 mm × 4.6 mm, 5 μm) by Agilent Technologies Inc. (Santa Clara, CA, USA), as earlier suggested by Gkioni et al. [44]. The chromatographic setup consisted of a 1260 Infinity II HPLC system with a diode array detector (DAD, 190–640 nm) and a manual injection valve, all from Agilent Technologies Inc. The column was maintained at a temperature of 30 °C, and the mobile phase flowed at 0.7 mL/min. Four solvents were utilized in the elution process: A (water with 0.1% formic acid), B (methanol), C (acetonitrile), and D (methanol/acetone, 80:20, *v*/*v*). The elution gradient, with solvent ratios A/B/C/D at specific time intervals, was as follows: 0 min: 55/5/40/0; 3 min: 5/5/90/0; 11 min: 5/5/90/0; 17 min: 0/10/90/0; 21 min: 0/10/90/0; 22 min: 0/0/0/100; and 45 min: 0/0/0/100. Data processing was completed using OpenLab Chemstation software, Version C (Agilent Technologies Inc., Santa Clara, CA, USA).

### 4.2. Preparation of Nanostructured Lipid Carriers

Nanostructured lipid carriers incorporating *T. lutea* extract (NLC-TLs) at a theoretical (initial) concentration of 20 mg/ mL were prepared. Additionally, empty nanoparticles (NLCs) were also prepared for comparison. Three independent batches were prepared for both NLCs and NLC-TLs. The composition of the samples is summarized in Table 1 [27,31].

The preparation of the nanoparticles was conducted using the hot homogenization method followed by ultrasonication. Initially, a conventional emulsion was formed by heating (65–70 °C) the aqueous and lipid phases and adding the aqueous phase to the lipid phase dropwise, while continuously stirring (200–300 rpm) using a thermal/magnetic stirrer (ARE, Velp Scientifica srl, Usmate Velate (mb), Italy). For the preparation of NLC-TL, the appropriate amount of *T. lutea* extract was accurately weighed to achieve a concentration of 20 mg/ mL and added to the melted lipid phase before the emulsification process. The same procedure was followed for the preparation of NLCs, omitting the addition of the extract and replacing its content with water.

The emulsion was cooled under stirring using a magnetic stirrer until it reached room temperature (RT). The dispersed particles were downsized by ultrasonication (Probe Sonicator Vibracell VCX130 PB, Sonics & Materials, Inc., Newtown, CT, USA) for 1 min/4 mL at an amplitude of 83%. The samples were cooled to room temperature under stirring (Vortex-Genie 2, Scientific Industries, Bohemia, NY, USA). The sonication and cooling process was repeated, and the samples were stored at 4 °C protected from light.

### 4.3. Physicochemical Characterization of Nanoparticles

#### 4.3.1. Particle Size and ζ-Potential Measurement

The determination of the mean particle size and the polydispersity index (PdI) and ζ-potential of NLCs and NLC-TLs was performed using Dynamic and Electrophoretic Light Scattering (DLS, ELS) on a Zetasizer Nano-ZS (Malvern Instruments Ltd., Malvern, UK) equipped with a He-Ne laser beam with a wavelength of 633 nm at a scattering angle of 173°. The nanoparticle dispersions were diluted with distilled water at a ratio of 1:6, and 0.6 mL of each diluted sample was transferred to a cuvette (DTS1070, Malvern Instruments Ltd., Malvern, UK). The reported size values were obtained from three independent measurements for each sample [31].

#### 4.3.2. Determination of Encapsulation and Loading of *T. lutea* Extract (TL) into Nanoparticles

To assess the efficiency of encapsulation and loading of TL into nanoparticles, it was essential to first remove any unincorporated fraction by size-exclusion chromatography using Sephadex G-75 (Sigma-Aldrich, Darmstadt, Germany) as the stationary phase and deionized water as the mobile phase.

An aliquot of 100 mL of each batch of NLC-TL was introduced into the column, and the eluate containing the entrapped *T. lutea* extract, characterized by its characteristic green color, was collected. The collected fractions from each batch were subjected to lyophilization after being stored at −80 °C overnight. Upon completion of the lyophilization process, each sample was dissolved in 1 mL of ethanol (EtOH). Additionally, 100 mL of the initial sample (prior to column passage) was diluted in EtOH to a total volume of 1 mL. The quantification of TL was performed based on its chlorophyll content. The absorbances of these samples at 655 nm were measured using a UV–Vis spectrophotometer (UV-1800 UV–Vis Spectrophotometer, SHIMADZU, Kyoto, Japan) [30]. The respective concentrations were calculated by preparing standard solutions of TL in EtOH and constructing a calibration curve.

The encapsulation efficiency (% EE) and loading (% LC) of the TL into nanoparticles were calculated using Equations (1) and (2), respectively:

(1) $$\%\mathrm{EE}=\frac{\mathrm{mass}\mathrm{of}\mathrm{loaded}\;T.\;lutea\;extract}{mass\;of\;initial\;T.\;lutea\;extract}\times 100$$

(2) $$\%LC=\frac{mass\;of\;loaded\;TL}{\mathrm{mass}\mathrm{of}\mathrm{carrier}+mass\;of\;loaded\;TL}\times 100$$

### 4.4. Stability Studies of Nanoparticles

#### 4.4.1. Centrifugation Test

Immediately after their preparation, the nanoparticles were subjected to centrifugation (Centrifuge Hermle Z32HK, Hermle Labortechnik GmbH, Wehingen, Germany). A volume of 1 mL from each sample was placed into Eppendorf tubes and centrifuged at 25 °C at a speed of 5000 rpm for 20 min. At the end of the test, all samples were macroscopically inspected for potential phase separation and their mean size, PDI, and ζ-potential were assessed.

#### 4.4.2. Accelerated Aging Test

All samples, one day after their preparation, were subjected to an accelerated aging test. This test comprised three cycles of heating at 40 °C and 75% relative humidity (RH) and cooling at 25 °C. At the end of the test (7th day), the size, PdI, and ζ-potential were assessed.

#### 4.4.3. Storage Under Various Temperature and Humidity Conditions

The colloidal stability of the dispersions was evaluated after their storage at 4 °C, 25 °C, and 40 °C, and 75% RH (HPP260, Memmert GmbH + Co. KG, Büchenbach, Germany), in light-protected containers. Measurements of their size, PdI, and ζ-potential were taken at predetermined time intervals (1, 8, 15, 22, and 30 days) over 30 days.

### 4.5. Study of the Antioxidant Activity of NLC-TLs

Free radical scavenging activity was assessed using the 2,2-diphenyl-1-picrylhydrazyl (DPPH) method [46,47,48].

A 0.1 mM solution of DPPH in methanol was prepared. To 195 mL of this DPPH solution that was placed in each well, various concentrations of NLCs, NLC-TLs, and *T. lutea* extract were added ranging from 10 to 2.5 mg/ mL, or standard solution. The final reaction mixtures were prepared in a 96-well plate format. The reaction mixtures were incubated at a constant temperature of 25 °C for 30 min protected from light. Following incubation, the absorbance of the reaction mixtures was measured at 540 nm using an absorbance microplate reader (SunriseTM TECAN Trading AG, Männedorf, Switzerland). Each sample was analyzed in triplicate.

The obtained absorbance values were recorded and analyzed using Microsoft Office Excel 2007 (Redmond, WA, USA). The percentage inhibition of DPPH radicals was calculated using the formula:

DPPH radical scavenging % = 100 − (Abs(control) − Abs(sample))/Abs(control) × 100, (3)

where Abs(sample) represents the absorbance of the reacted mixture of DPPH with the extract sample, and Abs(control) represents the absorbance of the DPPH solution.

Ascorbic acid was used as a positive control at concentrations ranging from 2 to 0.005 mg/mL to validate the efficacy of the assay.

All experiments were performed in triplicate to ensure the reliability and reproducibility of the results.

### 4.6. In Vitro SPF Assessment

To assess the SPF in vitro, 100 μL of NLC and NLC-TL were diluted to a final volume of 1 mL by adding ethanol to a final concentration of 2 mg/mL. An ethanol solution of TL with concentrations equivalent to that of the NLC-TL were also prepared. The absorbance of each sample in the range of 200–400 nm was recorded using a UV–Vis spectrophotometer (UV-1800 UV–Vis Spectrophotometer, SHIMADZU, Kyoto, Japan), and the SPF was calculated according to the Mansur equation (Equation (3)) [49]:

(4) $${SPF}_{spectophotometric}=CF\times \sum_{290}^{320}EE(\lambda )\times Ι(\lambda )\times Abs(\lambda )$$

where EE(*λ*): erythema action spectrum, I(*λ*): intensity of incident radiation for each wavelength, Abs(*λ*): absorption of the sunscreen product for each wavelength, and CF: correction factor (10). The values of EE × I are constant and were determined by Sayre et al., as presented in Table 2 [50].

### 4.7. Cell Viability Assay

Normal skin fibroblasts (NIH/3T3, ATCC, Manassas, VA, USA, #CRL-1658) were cultured in high-glucose DMEM supplemented with 10% Fetal Bovine Serum (FBS) and 1× Antibiotic-Antimycotic solution, under standard culturing conditions (37 °C, 5% CO_2,_ and 100% humidity). Cells were cultured in 24-well plates, washed once with PBS 1×, and starved for 16 h. The starvation medium consisted of DMEM supplemented with antibiotics. Cells were treated with five different concentrations of TL for 24 h. Then, 50 μL MTT stock solution (5 mg/mL in PBS) (50 μL in 500 μL medium/well) was added and incubated for 2 h at 37 °C. Plates were shaken for 1 min and the absorbance was defined by using a multi-plate reader at 450 nm (microplate reader).

### 4.8. Wound Healing Assay

After trypsinization, 15 × 10^4^ cells/well were seeded in 12-well plates and grown in a monolayer for 24 h. Then, cells were washed once with PBS 1× and starved for 2 h. A sterile plastic cell scratcher with 1 mm width was used. After the scratch, any detached cells were removed by washing them once with 1 mL PBS 1×. Then, 1 mL of starvation medium containing either the TL, NLC, NLC-TL, or the diluent (CTL) was added. For the wound healing assay, we used 10 μg/mL of TL and NLC-TL concentration corresponding to 10 μg/mL of TL. Photos were taken by using a micro-camera (AmScope, Irvine, CA, USA) at 400× magnification and 2048 × 1536 resolution at predefined time points (0 h, 24 h, and 48 h). The area corresponding to the scratch was defined by using the ImageJ wound healing size tool [51]. The % wound closure was defined by the following equation: (%) Wound Closure = (Area_(t0)_ − Area_(t)_)/Area_(t0)_ × 100%.

### 4.9. Investigation of the Influence of CEL and NEL on Skin Hydration

The impact of NLCs and NLC-TLs topically applied to the skin was assessed in vivo, employing non-invasive methodologies, as delineated in previous studies [9,52]. Ten healthy volunteers, ranging from 20 to 60 years of age, were recruited for this investigation after providing informed consent. Exclusion criteria included a history of skin conditions such as burns, wounds, allergies, or diseases, as well as the use of medications or pregnancy. Volunteers were instructed to refrain from using skincare products or washing the volar forearms, where the experimentation would occur. Each participant had three distinct squares (3 cm × 3 cm) marked on their left or right forearm. NLC and NLC-TL were applied on one square each at a dose of 2 mg/cm^2^, while one square was left untreated. The skin hydration on each square was measured before and at 60 and 120 min after application, using a Corneometer CM 825 (Courage+Khazaka electronic GmbH, Köln, Germany).

### 4.10. Statistical Analysis

The results were presented as the mean ± standard deviation (SD) of the measurements performed in three independent replicates. The statistical significance of the differences arising from the comparison of the mean values was determined using the *t*-test (Microsoft Office 365 Excel, Redmond, WA, USA). The significance level was set at *p* < 0.05.

For the wound healing assay, statistical analysis was performed with the GraphPad Prism 8 software (GraphPad Software, Boston, MA, USA). One-way ANOVA was used to compare the means of more than two independent groups of normally distributed data. *p*-value < 0.05 was considered statistically significant.

## 5. Conclusions

Overall, the encapsulation of *Tisochrysis lutea* extract in NLCs enhances the radical scavenging activity, wound healing properties, and skin hydration, demonstrating the potential of this formulation for cosmetic and therapeutic applications. The study does not only confirm the benefits of using NLCs as delivery systems for bioactive compounds but also suggests new features for the development of multifunctional skincare products incorporating marine-derived extracts for cosmetic and therapeutic applications, particularly in the fields of dermatology and wound care. Future research should focus on exploring the scalability of this formulation for commercial applications, and potentially combining the TL with other UV-blocking agents to enhance its SPF efficacy. Additionally, studies on other microalgae-derived compounds encapsulated in NLCs could provide further insights into the versatility and potential of this technology in various skincare and pharmaceutical applications.

## Figures and Tables

**Table 1:** The formula of NLCs and NLC-TLs.

Ingredient	INCI	Content (% *w*/*w*)	
		NLC	NLC-TL.
Saboderm TCC	Caprylic/Capric triglyceride	0.8	0.8
Softisan 100	Hydrogenated coco-glycerides	1.7	1.7
Emulmetik 900	Lecithin	2.5	2.5
Solutol HS 15	Macrogol (15)-hydroxystearate	1.0	1.0
*T. lutea* extract	*Tisochrysis lutea* extract	-	2.0
Purified water	Aqua	94.0	92.0

**Table 2:** Coefficients used in Mansur’s formula for calculation of SPF spectrophotometry [50].

Wave Length *λ* (nm)	EE × I
290	0.0150
295	0.0817
300	0.2874
305	0.3278
310	0.1864
315	0.0839
320	0.0180
Total	1

## Data Availability

Informed consent was obtained from all subjects involved in the study. The original contributions presented in the study are included in the article/Supplementary Materials; further inquiries can be directed to the corresponding authors.

## Supplementary Materials

The following supporting information can be downloaded at: https://www.mdpi.com/article/10.3390/md22110487/s1, Table S1. Physicochemical stability study of NLC and NLC-TL, monitoring their mean size, polydispersity index (PDI), and ζ-potential over time. Table S2. SPF estimation in vitro of NLC, NLC-TL, and TL according to the Mansur equation. Figure S1. (A). HPLC-DAD chromatogram at 280 nm (blue line) and at 430 nm (red line) from 0 to 20 min. The major xanthopyll is fucoxanthin. Other ingredients in this time frame: 1. Phenolic acid derivative (λmax: 210, 275 nm), 2. Phenolic acid derivative (λmax: 205, 265 nm), 3. Allenic *trans*-xanthophyll (λmax: 420,440,475 nm), 4. *trans*-xanthophyll (λmax: 450 nm), 5. Allenic *trans*-xanthophyll (λmax: 420,445,475 nm), 6. Allenic *cis*-xanthophyll (λmax: 310, 330,410,445,475 nm), 7. *trans*-xanthophyll (λmax: 405,425,465 nm), 8. *trans*-xanthophyll (λmax: 400,425,465 nm), 9. allenic *trans*-xanthophyll (λmax: 425,450,480 nm), and 10. *cis*-xanthophyll (λmax: 320,340,420,440,475 nm). (B). HPLC-DAD chromatogram at 430 nm (blue line) and at 639 nm (red line) after 20 min. The major ingredients are chlorophyll a, phaeophytin, and β-carotene. The DAD detector in our experimental setup scans from 190 to 640 nm, so the characteristic maximum of chlorophylls at ~665 nm could not be recorded. Figure S2. Absorbance spectra of NLC and NLC-TL (a) and *T. Lutea* extr. (b) at 200–400 nm.

## References

[1] Vázquez-Romero B., Perales J.A., Pereira H., Barbosa M., Ruiz J. (2022). Techno-economic assessment of microalgae production, harvesting and drying for food, feed, cosmetics, and agriculture. Sci. Total Environ..

[2] Anusree M.K., Leela K.M., Raj S., Sreenikethanam A., Bajhaiya A.K., Meena S.N., Nandre V., Kodam K., Meena R.S. (2023). Chapter 14—Marine microalgae: An emerging source of pharmaceuticals and bioactive compounds. Progress in Biochemistry and Biotechnology, New Horizons in Natural Compound Research.

[3] Kim S.Y., Kwon Y.M., Kim K.W., Kim J.Y.H. (2021). Exploring the Potential of *Nannochloropsis* sp. Extract for Cosmeceutical Applications. Mar. Drugs.

[4] Hong S.C., Yu H.S., Kim J.W., Lee E.H., Pan C.H., Hong K.W., Kim J.C. (2022). Protective effect of *Tisochrysis lutea* on dry eye syndrome via NF-κB inhibition. Sci. Rep..

[5] Bigagli E., D’Ambrosio M., Cinci L., Niccolai A., Biondi N., Rodolfi L., Dos Santos Nascimiento L.B., Tredici M.R., Luceri C. (2021). Comparative In Vitro Evaluation of the Anti-Inflammatory Effects of a *Tisochrysis lutea* Extract and Fucoxanthin. Mar. Drugs.

[6] Gao F., Teles Cabanelas I.I., Ferrer-Ledo N., Wijffels R.H., Barbosa M.J. (2020). Production and high throughput quantification of fucoxanthin and lipids in *Tisochrysis lutea* using single-cell fluorescence. Bioresour. Technol..

[7] Lina R., Lepine O., Jaouen P., Masse A. (2022). Recovery of Water-Soluble Compounds from *Tisochrysis lutea*. Membranes.

[8] Andriopoulos V., Gkioni M.D., Koutra E., Mastropetros S.G., Lamari F.N., Hatziantoniou S., Kornaros M. (2022). Total Phenolic Content, Biomass Composition, and Antioxidant Activity of Selected Marine Microalgal Species with Potential as Aquaculture Feed. Antioxidants.

[9] Liakopoulou A., Mountsaki S., Andriopoulos V., Gkioni M., Kornaros M., Lamari F., Hatziantoniou S. (2022). Study of the cosmetic properties of microalgae extracts—The case of *Nannochloropsis oculata*. Rev. Clin. Pharmacol. Pharmacokinet. Int. Ed..

[10] Pereira H., Sá M., Maia I., Rodrigues A., Teles I., Wijffels R.H., Navalho J., Barbosa M. (2021). Fucoxanthin production from *Tisochrysis lutea* and *Phaeodactylum tricornutum* at industrial scale. Algal Res..

[11] Gonçalves de Oliveira-Júnior R., Grougnet R., Bodet P.E., Bonnet A., Nicolau E., Jebali A., Rumin J., Picot L. (2020). Updated pigment composition of *Tisochrysis lutea* and purification of fucoxanthin using centrifugal partition chromatography coupled to flash chromatography for the chemosensitization of melanoma cells. Algal Res..

[12] Endo H., Hanawa Y., Araie H., Araie H., Suzuki I., Shiraiwa Y. (2018). Overexpression of *Tisochrysis lutea* Akd1 identifies a key cold-induced alkenone desaturase enzyme. Sci. Rep..

[13] Custódio L., Soares F., Pereira H., Barreira L., Vizetto-Duarte C., Rodrigues M.J., Rauter A.P., Alberício F., Varela J. (2014). Fatty acid composition and biological activities of *Isochrysis galbana* T.-ISO, *Tetraselmis* sp. and *Scenedesmus* sp.: Possible application in the pharmaceutical and functional food industries. J. Appl. Phycol..

[14] Ryckebosch E., Bruneel C., Termote-Verhalle R., Goiris K., Muylaert K., Foubert I. (2014). Nutritional evaluation of microalgae oils rich in omega-3 long chain polyunsaturated fatty acids as an alternative for fish oil. Food Chem..

[15] Andriopoulos V., Lamari F.N., Hatziantoniou S., Kornaros M. (2022). Production of Antioxidants and High Value Biomass from Nannochloropsis oculata: Effects of pH, Temperature and Light Period in Batch Photobioreactors. Mar. Drugs.

[16] Mayer C., Richard L., Côme M., Ulmann L., Nazih H., Chénais B., Ouguerram K., Mimouni V. (2021). The Marine Microalga, *Tisochrysis lutea*, Protects against Metabolic Disorders Associated with Metabolic Syndrome and Obesity. Nutrients.

[17] Bigagli E., Cinci L., Niccolai A., Biondi N., Rodolfi L., D’Ottavio M., D’Ambrosio M., Lodovici M., Tredici M.R., Luceri C. (2018). Preliminary data on the dietary safety, tolerability and effects on lipid metabolism of the marine microalga *Tisochrysis lutea*. Algal Res..

[18] Iglesias M.J., Soengas R., Probert I., Guilloud E., Gourvil P., Mehiri M., López Y., Cepas V., Gutiérrez-Del-Río I., Redondo-Blanco S. (2019). NMR characterization and evaluation of antibacterial and antiobiofilm activity of organic extracts from stationary phase batch cultures of five marine microalgae (*Dunaliella* sp., *D. salina*, *Chaetoceros calcitrans*, *C. gracilis* and *Tisochrysis lutea*). Phytochemistry.

[19] Rodríguez-Luna A., Ávila-Román J., González-Rodríguez M.L., Cózar M.J., Rabasco A.M., Motilva V., Talero E. (2018). Fucoxanthin-Containing Cream Prevents Epidermal Hyperplasia and UVB-Induced Skin Erythema in Mice. Mar. Drugs.

[20] Mu L., Sprando R.L. (2010). Application of Nanotechnology in Cosmetics. Pharm. Res..

[21] Nguyen T.-T.-L., Duong V.-A. (2022). Solid Lipid Nanoparticles. Encyclopedia.

[22] Oliveira C., Coelho C., Teixeira J.A., Ferreira-Santos P., Botelho C.M. (2022). Nanocarriers as Active Ingredients Enhancers in the Cosmetic Industry—The European and North America Regulation Challenges. Molecules.

[23] Salvioni L., Morelli L., Ochoa E., Labra M., Fiandra L., Palugan L., Prosperi D., Colombo M. (2021). The emerging role of nanotechnology in skincare. Adv. Colloid Interface Sci..

[24] Thomas L.M., Khasraghi A.H. (2020). Nanotechnology-Based Topical Drug Delivery Systems for Management of Dandruff and Seborrheic Dermatitis: An overview. Iraqi J. Pharm. Sci..

[25] Subramaniam B., Siddik Z.H., Nagoor N.H. (2020). Optimization of nanostructured lipid carriers: Understanding the types, designs, and parameters in the process of formulations. J. Nanoparticle Res..

[26] Souto E.B., Baldim I., Oliveira W.P., Rao R., Yadav N., Gama F.M., Mahant S. (2020). SLN and NLC for topical, dermal, and transdermal drug delivery. Expert Opin. Drug Deliv..

[27] Liakopoulou A., Mourelatou E., Hatziantoniou S. (2021). Exploitation of traditional healing properties, using the nanotechnology’s advantages: The case of curcumin. Toxicol. Rep..

[28] Gallego R., Tardif C., Parreira C., Guerra T., Alves J., Ibáñez E., Herrero M. (2020). Simultaneous extraction and purification of fucoxanthin from *Tisochrysis lutea* microalgae using compressed fluids. J. Sep. Sci..

[29] Yuan K., Sun Y., Liang F., Pan F., Hu M., Hua F., Yuan Y., Nie J., Zhang Y. (2022). Tyndall-effect-based colorimetric assay with colloidal silver nanoparticles for quantitative point-of-care detection of creatinine using a laser pointer pen and a smartphone. RSC Adv..

[30] Lichtenthaler H.Κ., Wellburn A.R. (1983). Determinations of total carotenoids and chlorophylls a and b of leaf extracts in different solvents. Biochem. Soc. Trans..

[31] Flekka K., Dimaki V.D., Mourelatou E., Avgoustakis K., Lamari F.N., Hatziantoniou S. (2024). Stability and Retention of Nanoemulsion Formulations Incorporating Lavender Essential Oil. Cosmetics.

[32] Gordillo-Galeano A., Mora-Huertas C.E. (2021). Hydrodynamic diameter and zeta potential of nanostructured lipid carriers: Emphasizing some parameters for correct measurements. Colloids Surf. A Physicochem. Eng..

[33] Ashfaq R., Rasul A., Asghar S., Kovács A., Berkó S., Budai-Szűcs M. (2023). Lipid Nanoparticles: An Effective Tool to Improve the Bioavailability of Nutraceuticals. Int. J. Mol. Sci..

[34] Yang J., Xiong L., Li M., Xiao J., Geng X., Wang B., Sun Q. (2019). Preparation and Characterization of Tadpole- and Sphere-Shaped Hemin Nanoparticles for Enhanced Solubility. Nanoscale Res. Lett..

[35] Bera B., Khazal R., Schroën K. (2021). Coalescence dynamics in oil-in-water emulsions at elevated temperatures. Sci. Rep..

[36] Gonçalves C., Ramalho M.J., Silva R., Silva V., Marques-Oliveira R., Silva A.C., Pereira M.C., Loureiro J.A. (2021). Lipid Nanoparticles Containing Mixtures of Antioxidants to Improve Skin Care and Cancer Prevention. Pharmaceutics.

[37] Zerres S., Stahl W. (2020). Carotenoids in human skin. Biochimica et biophysica acta. Mol. Cell Biol. Lipids.

[38] Ordoudi S.A., Befani C.D., Nenadis N., Koliakos G.G., Tsimidou M.Z. (2009). Further examination of antiradical properties of Crocus sativus stigmas extract rich in crocins. J. Agric. Food Chem..

[39] Elkhateeb O., Badawy M.E.I., Tohamy H.G., Abou-Ahmed H., El-Kammar M., Elkhenany H. (2023). Curcumin-infused nanostructured lipid carriers: A promising strategy for enhancing skin regeneration and combating microbial infection. BMC Vet. Res..

[40] COLIPA GUIDLINES, International Sun Protection Factor (SPF) Test Method. https://downloads.regulations.gov/FDA-1978-N-0018-0698/attachment_65.pdf.

[41] Garzarella K., Caswell M. (2013). Disparate SPF testing methodologies generate similar SPFs. J. Cosmet. Sci..

[42] Santiesteban-Romero B., Martínez-Ruiz M., Sosa-Hernández J.E., Parra-Saldívar R., Iqbal H.M.N. (2022). Microalgae Photo-Protectants and Related Bio-Carriers Loaded with Bioactive Entities for Skin Applications—An Insight of Microalgae Biotechnology. Mar. Drugs.

[43] Patel P., Garala K., Singh S., Prajapati B.G., Chittasupho C. (2024). Lipid-Based Nanoparticles in Delivering Bioactive Compounds for Improving Therapeutic Efficacy. Pharmaceuticals.

[44] Gkioni M.D., Andriopoulos V., Koutra E., Hatziantoniou S., Kornaros M., Lamari F.N. (2022). Ultrasound-Assisted Extraction of Nannochloropsis oculate with Ethanol and Betaine: 1,2-Propanediol Eutectic Solvent for Antioxidant Pigment-Rich Extracts Retaining Nutritious the Residual Biomass. Antioxidants.

[45] Lichtenthaler H.K., Buschmann C. (2001). Chlorophylls and Carotenoids: Measurement and Characterization by UV-VIS Spectroscopy. Curr. Protoc. Food Anal. Chem..

[46] Brand-Williams W., Cuvelier M.E., Berset C. (1995). Use of a free radical method to evaluate anioxidant activity. LWT-Food Sci. Technol..

[47] Kim D.O., Lee K.W., Lee H.J., Lee C.Y. (2002). Vitamin C equivalent antioxidant capacity (VCEAC) of phenolic phytochemicals. J. Agric. Food Chem..

[48] Siakavella I.K., Lamari F., Papoulis D., Orkoula M., Gkolfi P., Lykouras M., Avgoustakis K., Hatziantoniou S. (2020). Effect of Plant Extracts on the Characteristics of Silver Nanoparticles for Topical Application. Pharmaceutics.

[49] Mansur J.S., Breder M.N.R., Mansur M.C.A., Azulay R.D. (1989). Determinaçäo do fator de proteçäo solar por espectrofotometria. Bras Dermatol Rio De Jan..

[50] Sayre R.M., Agin P.P., LeVee G.J., Marlowe E. (1979). A comparison of in vivo and in vitro testing of sunscreening formulas. Photochem Photobiol..

[51] Rasband W.S. National Institutes of Health: Bethesda, MD, USA, 1997–2018. https://imagej.nih.gov/ij/.

[52] Wolf M., Klang V., Stojcic T., Fuchs C., Wolzt M., Valenta C. (2018). NLC versus nanoemulsions: Effect on physiological skin parameters during regular in vivo application and impact on drug penetration. Int. J. Pharm..

