# Supplementary material for: Encapsulation of Microalgae Tisochrysis lutea Extract in Nanostructured Lipid Carriers (NLCs) and Evaluation of Their Sunscreen, Wound Healing, and Skin Hydration Properties
Source: Mar Drugs. 2024 Oct 30;22(11):487. doi: 10.3390/md22110487 (PMC11595965; doi:10.3390/md22110487)
Supplement: Supplementary file 1 [file marinedrugs-22-00487-s001.zip › marinedrugs-3269382-supplementary.pdf]

supplementary

Encapsulation of microalgae *Tisochrysis lutea* extract in Nanostructured Lipid Carriers (NLCs) and evaluation of their sunscreen, wound healing and skin hydration properties.

Natalia Chatzopoulou [a], Chrysi Chaikali [a], Eleni Mourkogianni [b], Constantinos M. Mikelis [b], Vasilis Andriopoulos [d], Michael Kornaros [d], Konstantinos Avgoustakis [a], Fotini Lamari [c], Sophia Hatziantoniou [a*]

[a] Laboratory of Pharmaceutical Technology, [b] Laboratory of Molecular Pharmacology, [c] Laboratory of Pharmacognosy and Chemistry of Natural Products, Dept of Pharmacy, School of Life Sciences, University of Patras, 26 504, Rion, Greece

[d] Laboratory of Biochemical Engineering & Environmental Technology (LBEET), Department of Chemical Engineering, University of Patras, 26504 Patras, Greece

Table S1. Physicochemical stability study of NLC and NLC-TL, monitoring their mean size, polydispersity index (PDI) and ζ-potential over time.

NLC	time (d)	Mean size (nm)	PDI	ζ-potential (mV)
After preparation	1	99,83 ± 4,01	0,264 ± 0,004	-46,9 ± 1,1
Centrifugation	After	102,25 ± 5,61	0,261 ± 0,009	-46,6 ± 2,96
Accelerated aging	After	82,90 ± 4,52	0,223 ± 0,023	-26,4 ± 6,2
4 ºC	8	83,98 ± 2,20	0,232 ± 0,018	-31,9 ± 2,4
	15	98,37 ± 6,99	0,276 ± 0,014	-39,5 ± 6,2
	22	90,33 ± 3,73	0,262 ± 0,003	-36,5 ± 2,4
	30	103,25 ± 5,28	0,278 ± 0,014	-49,8 ± 5,5
25 ºC	8	89,74 ± 12,27	0,263 ± 0,084	-29,3 ± 4,7
	15	96,02 ± 3,88	0,281 ± 0,019	-38,3 ± 6,3
	22	90,44 ± 0,16	0,256 ± 0,004	-34,4 ± 8,2
	30	101,2 ± 3,19	0,301 ± 0,018	-35,3 ± 7,0
40 ºC / 75% RH	8	84,85 ± 3,46	0,213 ± 0,062	-31,3 ± 5,8
	15	93,23 ± 7,64	0,294 ± 0,084	-31,7 ± 6,8
	22	56,11 ± 12,51	0,331 ± 0,128	-31,4 ± 8,4
	30	172,49 ± 205,7	0,399 ± 0,162	-39,2 ± 5,9
**NLC-TL**	**time (d)**	**Mean size (nm)**	**PDI**	**ζ-potential (mV)**
After preparation	1	103,88 ± 7,14	0,255 ± 0,003	-46,7 ± 7,4
Centrifugation	After	106,21 ± 9,14	0,274 ± 0,03	-48,0 ± 4,4
Accelerated aging	After	99,07 ± 10,39	0,299 ± 0,071	-42,6 ± 7,8
4 ºC	8	108,77 ± 4,27	0,269 ± 0,018	-48,6 ± 1,4
	15	103,52 ± 3,55	0,270 ± 0,017	-50,2 ± 1,5
	22	104,07 ± 3,01	0,274 ± 0,010	-48,3 ± 0,9
	30	103,63 ± 3,72	0,265 ± 0,005	-50,4 ± 2,5
25 ºC	8	108,97 ± 3,45	0,273 ± 0,006	-50,3 ± 4,0
	15	98,90 ± 5,03	0,258 ± 0,004	-49,8 ± 4,7
	22	103,10 ± 1,51	0,323 ± 0,120	-44,0 ± 5,3

	30	102,46 ± 6,92	0,268 ±0,010	-44,9 ± 3,5
40 ^{o}C / 75% RH	8	107,64 ± 9,10	0,324 ± 0,042	-52,0 ± 4,2
	15	94,58 ± 7,09	0,268 ± 0,016	-49,0 ± 2,8
	22	95,99 ± 6,58	0,322 ± 0,048	-41,8 ± 4,6
	30	106,10 ± 67,15	0,318 ± 0,104	-44,5 ± 2,2

Table S2. SPF estimation *in vitro* of NLC, NLC-Tl and *T. lutea* extract according to the Mansur Equation [49].

EE×I	Wavelength (nm)	NLC-TL	NLC	*T. lutea* extract
0.015	290	0.812	0.286	0,214
0.0817	295	0.794	0.244	0,190
0.2874	300	0.785	0.210	0,179
0.3278	305	0.754	0.182	0,170
0.1864	310	0.727	0.165	0,163
0.0839	315	0.714	0.156	0,160
0.018	320	0.696	0.149	0,158
**SPF**		**7.58 ±2.83**	**1.91 ±0.42**	**1.73 ± 0.37**

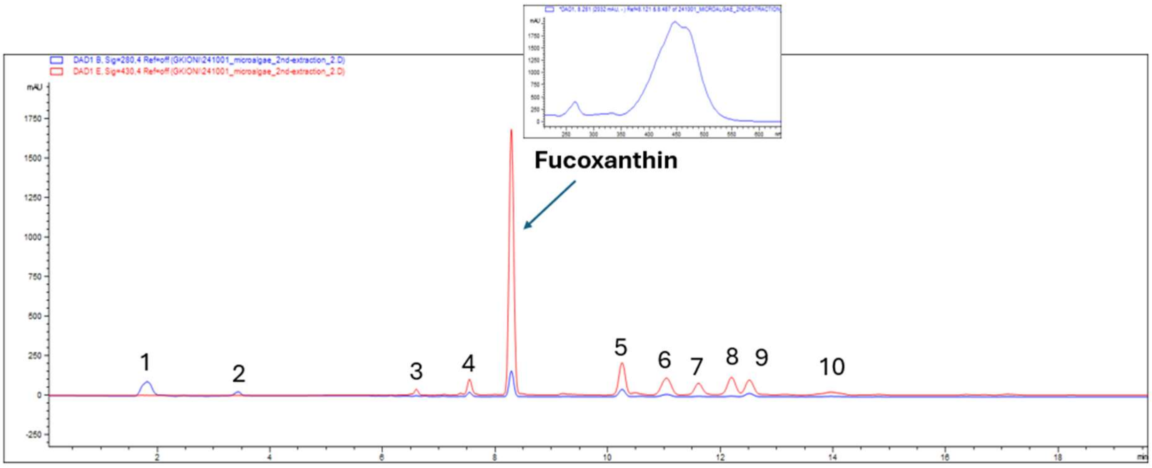

Figure S1. **A.** HPLC-DAD chromatogram at 280 nm (blue line) and at 430 nm (red line) from 0 to 20 min. The major xanthopyll is fucoxanthin. Other ingredients in this time frame: **1**. Phenolic acid derivative (λmax: 210, 275 nm), **2**. Phenolic acid derivative (λmax: 205, 265 nm), **3**. Allenic *trans*-xanthophyll (λmax: 420,440,475 nm), **4**. *trans*-xanthophyll (λmax: 450 nm), **5**. Allenic *trans*-xanthophyll (λmax: 420,445,475 nm), **6**. Allenic *cis*-xanthophyll (λmax: 310, 330,410,445,475 nm), **7**. *trans*-xanthophyll (λmax: 405,425,465 nm), **8**. *trans*-xanthophyll (λmax: 400,425,465 nm), **9**. allenic *trans*-xanthophyll (λmax: 425,450,480 nm), **10**. *cis*-xanthophyll (λmax: 320,340,420,440,475 nm)

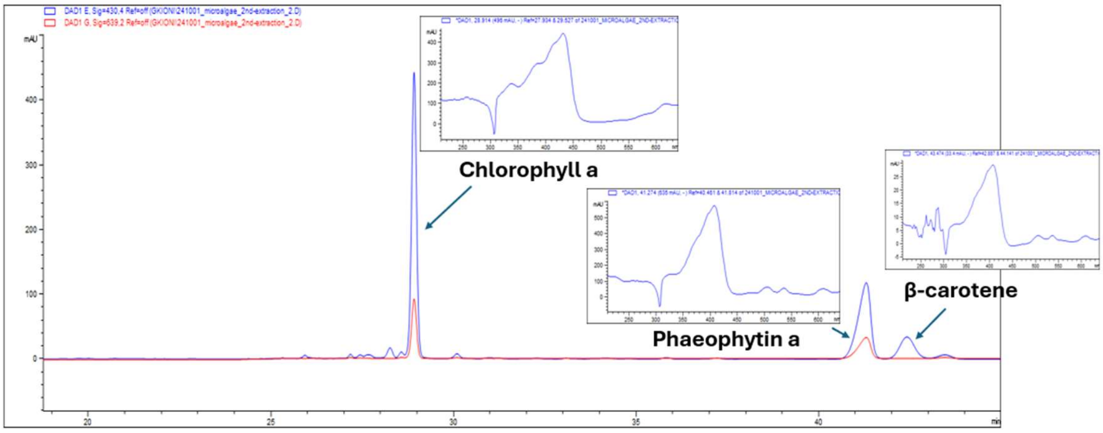

Figure S1. **B.** HPLC-DAD chromatogram at 430 nm (blue line) and at 639 nm (red line) after 20 min. The major ingredients are chlorophyll a, phaeophytin and β-carotene. The DAD detector in our experimental set-up scans from 190 to 640 nm, so the characteristic maximum of chlorophylls at ~665 nm could not be recorded.

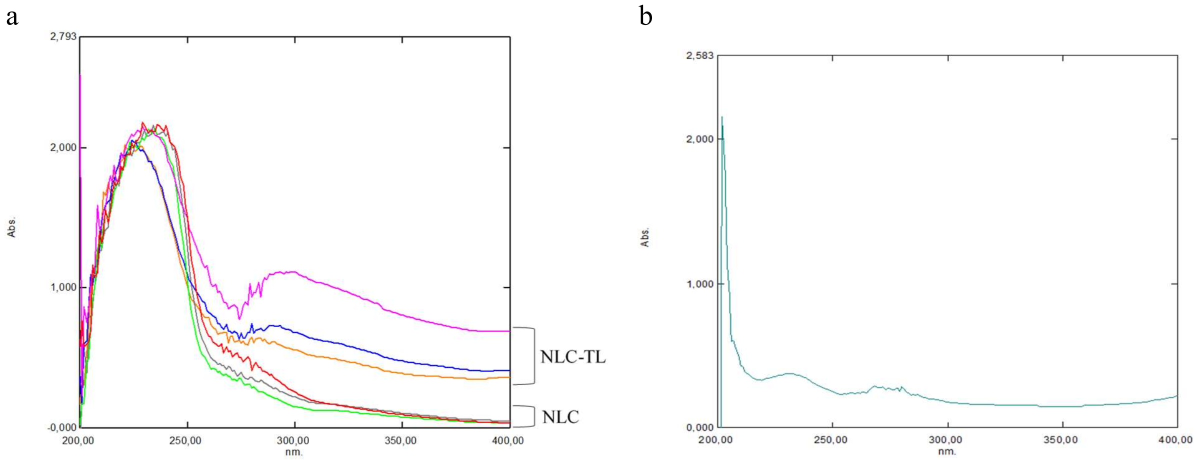

Figure S2. Absorbance spectra of NLC and NLC-TL a) and T. Lutea extr. b) at 200 – 400 nm.
